# Etiology of community-acquired pneumonia and diagnostic yields of microbiological methods: a 3-year prospective study in Norway

**DOI:** 10.1186/s12879-015-0803-5

**Published:** 2015-02-15

**Authors:** Jan C Holter, Fredrik Müller, Ola Bjørang, Helvi H Samdal, Jon B Marthinsen, Pål A Jenum, Thor Ueland, Stig S Frøland, Pål Aukrust, Einar Husebye, Lars Heggelund

**Affiliations:** Department of Internal Medicine, Vestre Viken Hospital Trust, Drammen, Norway; Department of Medical Microbiology, Vestre Viken Hospital Trust, Drammen, Norway; Department of Radiology, Vestre Viken Hospital Trust, Drammen, Norway; Research Institute of Internal Medicine, Oslo University Hospital Rikshospitalet, Oslo, Norway; Department of Microbiology, Oslo University Hospital Rikshospitalet, Oslo, Norway; Section of Clinical Immunology and Infectious Diseases, Oslo University Hospital Rikshospitalet, Oslo, Norway; Institute of Clinical Medicine, Faculty of Medicine, University of Oslo, Oslo, Norway; K.G. Jebsen Inflammatory Research Center, University of Oslo, Oslo, Norway; Department of Microbiology, Oslo University Hospital Ullevaal, Oslo, Norway; Department of Radiology, Hospital of Southern Norway HF, Kristiansand, Norway

**Keywords:** Community-acquired pneumonia, Etiology, Microbiology, Virology, Diagnosis

## Abstract

**Background:**

Despite recent advances in microbiological techniques, the etiology of community-acquired pneumonia (CAP) is still not well described. We applied polymerase chain reaction (PCR) and conventional methods to describe etiology of CAP in hospitalized adults and evaluated their respective diagnostic yields.

**Methods:**

267 CAP patients were enrolled consecutively over our 3-year prospective study. Conventional methods (i.e., bacterial cultures, urinary antigen assays, serology) were combined with nasopharyngeal (NP) and oropharyngeal (OP) swab samples analyzed by real-time quantitative PCR (qPCR) for *Streptococcus pneumoniae,* and by real-time PCR for *Mycoplasma pneumoniae, Chlamydophila pneumoniae, Bordetella pertussis* and 12 types of respiratory viruses.

**Results:**

Etiology was established in 167 (63%) patients with 69 (26%) patients having ≥1 copathogen. There were 75 (28%) pure bacterial and 41 (15%) pure viral infections, and 51 (19%) viral–bacterial coinfections, resulting in 126 (47%) patients with bacterial and 92 (34%) patients with viral etiology. *S. pneumoniae* (30%), influenza (15%) and rhinovirus (12%) were most commonly identified, typically with ≥1 copathogen. During winter and spring, viruses were detected more frequently (45%, *P*=.01) and usually in combination with bacteria (39%). PCR improved diagnostic yield by 8% in 64 cases with complete sampling (and by 15% in all patients); 5% for detection of bacteria; 19% for viruses (*P*=.04); and 16% for detection of ≥1 copathogen. Etiology was established in 79% of 43 antibiotic-naive patients with complete sampling. *S. pneumoniae* qPCR positive rate was significantly higher for OP swab compared to NP swab (*P*<.001). Positive rates for serology were significantly higher than for real-time PCR in detecting *B. pertussis* (*P*=.001) and influenza viruses (*P*<.001).

**Conclusions:**

Etiology could be established in 4 out of 5 CAP patients with the aid of PCR, particularly in diagnosing viral infections. *S. pneumoniae* and viruses were most frequently identified, usually with copathogens. Viral–bacterial coinfections were more common than pure infections during winter and spring; a finding we consider important in the proper management of CAP. When swabbing for qPCR detection of *S. pneumoniae* in adult CAP, OP appeared superior to NP, but this finding needs further confirmation.

**Trial registration:**

ClinicalTrials.gov Identifier: NCT01563315.

**Electronic supplementary material:**

The online version of this article (doi:10.1186/s12879-015-0803-5) contains supplementary material, which is available to authorized users.

## Background

Community-acquired pneumonia (CAP) is a common disease and a significant cause of morbidity and mortality worldwide [1-3]. Differences in epidemiology of pathogens make the knowledge of local etiology crucial for the appropriate choice of empirical antimicrobial treatment, which has a major impact on the prognosis of the patient [4-7]. An important rationale for microbial testing is to enable pathogen-directed therapy, and thus avoid unnecessary antibiotic use [8]. In clinical practice causative pathogens often remain unknown, and the majority of patients are treated empirically [8]. Moreover, published data on etiology of CAP in Norway [9], as well as in several other countries, are hampered by old data, limited samples and detection methods confined to bacterial culture.

The use of diagnostic methods based on polymerase chain reaction (PCR) have resulted in increased detection of many bacterial and viral pathogens associated with CAP [8]. One of the advantages of PCR is its ability to identify respiratory pathogens also after initiation of antibiotic therapy [10]. Some recent studies have shown that a high microbial yield can be achieved when real-time PCR assays are combined with conventional diagnostic methods like bacterial cultures, urinary antigen assays and serology [11-16]. Such extensive approaches have led to recognition of viruses as important causes of CAP and that coinfections are more common than previously thought [17,18].

The aim of this study was to describe etiology of CAP in adults admitted to a general hospital in South-Eastern Norway using conventional methods and real-time PCR assays for the detection of respiratory pathogens. We also investigated the diagnostic yields of the methods applied, especially the potential benefit of PCR.

## Methods

### Patients and study design

Adults (≥18 years old) with suspected pneumonia admitted to Medical Department, Drammen Hospital, Vestre Viken Health Trust—an acute care 270-bed general hospital in the province of South-Eastern Norway serving a mixed rural and urban population of about 160 000 inhabitants—were consecutively evaluated for inclusion in a prospective study from January 2008 to January 2011. CAP was defined as (i) presence of a new pulmonary infiltrate on chest radiograph, (ii) rectal temperature >38.0°C, and (iii) at least 1 of the following symptoms or signs: cough (productive or nonproductive), dyspnea, respiratory chest pain, crackles or reduced respiratory sounds. These criteria had to be present within 48 h of admission. Patients were excluded from the study when the chest radiographic examination showed noninfectious causes such as pulmonary infarction, tumor or bronchiectasis, or if the patient was discharged from the hospital within the 2 weeks prior to admission. All study participants were invited to an outpatient follow-up 6 weeks after hospital discharge, including chest radiography and a convalescent blood sample. Consent was obtained at the time of recruitment. The study was approved by the Regional Committee for Medical and Health Research Ethics in South-Eastern Norway (reference number: S-06266a).

### Clinical data collection

Clinical data were recorded on paper case record forms with missing data abstracted from medical records. Chest radiographic patterns were thoroughly examined by an independent experienced radiologist.

### Microbial sample collection

Blood cultures (BD BACTEC™ Blood Culture System, Sparks, Maryland, USA) were obtained prior to commencing antibiotic therapy. Clinicians at the emergency room or on the wards collected nasopharyngeal (NP) swab samples—and, when possible, sputum specimens for Loeffler stain, Gram stain (optional) and culture—also before initiation of antibiotic therapy. In some cases, these samples were collected at the time of recruitment within 48 h after hospital admission. An additional NP swab and oropharyngeal (OP) swab from posterior oropharynx and both tonsillar pillars were collected for PCR analysis on separate transport media (Copan Flocked Swabs and UTM-RT Transport Medium System, Brescia, Italy). Urine sample was collected for specific antigen detection. Bronchoalveolar lavage (BAL) and diagnostic thoracentesis were performed on medical indication as judged by the treating clinician, usually when the pneumonia did not resolve, or—in the case of thoracentesis—the cause of build up pleural fluid was not known. Acute-phase serum samples were collected at the time of recruitment. Convalescent-phase serum samples were obtained at the time of outpatient follow-up, approximately 6 weeks after hospital discharge.

### Conventional microbiological methods

Bacteriological specimens were cultured on standard media. BAL was cultured quantitatively in accordance with accepted methods [19]. Growth of specific pathogens detected in sputum and NP samples were judged semi-quantitatively as low, intermediate or abundant. The most purulent part of sputum samples was processed for microscopy. Only samples displaying >25 polymorphonuclear leukocytes and <10 squamous epithelial cells per 100× power field were considered acceptable for culture [20]. For serology, commercially available assays were used according to the manufacturer’s instructions for detection of *Mycoplasma pneumoniae*, *Chlamydophila pneumoniae*, *Bordetella pertussis,* and influenza A and B viruses (see Additional file 1). Urinary antigen detection tests for *Streptococcus pneumoniae* and *Legionella pneumophila* serogroup 1 were performed with the BinaxNOW pneumococcal urinary antigen test and the BinaxNOW Legionella urinary antigen test (Binax, ME, USA).

### PCR

All NP and OP samples were analyzed by real-time quantitative PCR (qPCR) for detection of *S. pneumoniae* using primers specific for the pneumolysin (*ply*) gene as described by Greiner et al. [21]; by real-time PCR for presence of bacterial (*M. pneumoniae*, *C. pneumoniae* and *B. pertussis*) and adenovirus DNA; and by real-time reverse-transcription PCR (RT-PCR) for detection of RNA viruses (influenza A and B viruses [reported as influenza viruses], and H1N1 on influenza A virus positive samples, parainfluenza viruses types 1–3 [reported as parainfluenza viruses], metapneumovirus, rhinovirus, enterovirus and respiratory syncytial virus (A and B) [reported as respiratory syncytial virus]). Detection of *S. pneumoniae* and respiratory viruses were performed retrospectively. Sputum samples and BAL were examined, if medically indicated, by use of real-time PCR detection of *L. pneumophila* and/or *Pneumocystis jirovecii*. Additional file 1 provide details of the real-time PCR methods and pneumococcal qPCR assay.

### Complete sample collection definition

A complete sample collection constituted the collection of blood, sputum and NP samples for culture; NP and OP samples analyzed for *S. pneumoniae, M. pneumoniae*, *C. pneumoniae, B. pertussis* and 12 types of respiratory viruses by use of PCR; serological testing for *M. pneumoniae*, *C. pneumoniae*, *B. pertussis,* and influenza A and B viruses; and urine antigen assays for detection of pneumococcal and *L. pneumophila* antigens.

### Classification of etiology

Etiology was considered to be *definite* if any of the following criteria were met: a microorganism was cultured from blood or pleural fluid; BAL yielded growth of >10^5^ colony-forming units per milliliter (cfu/mL) of one microbial species; urinary antigen test for *S. pneumoniae* or *L. pneumophila* was positive; or real-time PCR detection of *L. pneumophila* or *P. jirovecii* in sputum samples or BAL was positive.

Etiology was considered to be *probable* if any of the following criteria were met: bacteria cultured from sputum or NP swabs (at least intermediate growth was demanded for bacteria other than *S. pneumoniae* where any quantity of growth was accepted); real-time PCR detection of *M. pneumoniae, C. pneumoniae, B. pertussis* or a respiratory virus was positive; serological diagnosis of *M. pneumoniae*, *C. pneumoniae* or influenza viruses by CFT seroconversion (i.e., <10 to ≥20 or conversely), or a 4-fold titer rise or fall, or a high CFT titer (i.e., *M. pneumoniae* ≥160, *C. pneumoniae* ≥80, influenza A virus ≥160, influenza B virus ≥80) of either one or both of acute- and convalescent-phase samples, or presence of IgM antibodies ≥840 U/mL for *M. pneumoniae* or ≥1.1 cut-off-index for *C. pneumoniae*; or serological diagnosis of *B. pertussis* by a 2-fold rise of PT-IgG (to >30 IU/mL in convalescent-phase serum) or fall (from >30 IU/mL in acute-phase serum), or presence of PT-IgG ≥80 IU/mL in acute- and/or convalescent-phase samples [22,23]. Pneumococcal DNA from NP samples [24] corresponding to ≥10^5^ cfu/mL [25] by use of qPCR was also considered to be of probable significance. The same conclusions were drawn for OP samples based on the results from Principi et al. [26]. A case was considered coinfected if >1 pathogen, classified according to the above-mentioned criteria, was found.

### Statistical analysis

Data were analyzed using PASW statistics software, version 20.0 (IBM SPSS, Chicago, IL). Categorical data were summarized using frequency counts and percentages. Continuous data were presented as mean and standard deviation (SD). The McNemar’s test with Yates’ continuity correction for paired proportions and the kappa statistics were used to compare the different techniques with respect to the percentage with positive results, and to evaluate the degree of agreement, respectively. Methods were selected for comparison based on level of diagnostic significance according to the classification; i.e., only techniques of which a positive result would give the same level of significance (definite or probable) were compared. In case of zero cells, the value of 0.5 was applied to each cell of the table in order to be able to perform the calculations. Kappa value <0.20 is poor, 0.21–0.40 fair, 0.41–0.60 moderate, 0.61–0.80 good, and kappa value >0.81 is very good agreement. For other category variables, groups were compared by use of Pearson’s chi-square test or Fisher’s exact test. In all instances a *P* of < .05 (2-tailed) was considered statistically significant.

## Results

### Patients

**Figure 1:**
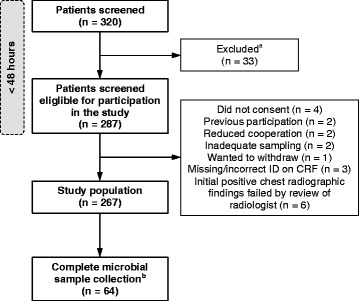
**Study flowchart.** ^a^Thirty-three patients were excluded due to no findings of new infiltrate (n = 19); chest radiograph was not performed (n = 1); fever was not documented (n = 4); noninfectious cause and/or bronchial obstruction (n = 7); or previous discharge from hospital within the last 14 days (n = 2). ^b^Of these, 43 patients had not received antibiotics prior to hospital admission. Abbreviations: ID, identification; CRF, case record form.

Of the 320 screened patients, 267 patients were included in the study (Figure 1). Median age was 66 years (range 19–100 years) (Table 1). The average stay in the hospital was 6.8 days (range 1–75 days). Forty-eight (18%) patients were admitted to intensive care unit. The overall 30-day mortality rate was 3.7% (i.e., 10 of 267 patients died).

**Table 1:** **Baseline characteristics of 267 hospitalized adult patients with community-acquired pneumonia**

**Characteristics**	**Patients**
Age, years	66 (52–78)
Female/male	127 (48)/140 (52)
Nursing home resident	4 (1.5)
Patients with any comorbidity	172 (64)
Atherosclerosis^a^	71 (27)
COPD	60 (23)
Immune disorder^b^	34 (13)
Diabetes mellitus	33 (12)
Renal disease	32 (12)
Asthma	25 (9)
Congestive heart failure	22 (8)
Neurological^c^	19 (7)
Dementia	15 (6)
Active malignancy	13 (5)
Innate or acquired immunodeficiency^d^	11 (4)
Liver disease	4 (1.5)
Active smoker	65 (24)
Travel history^e^	37 (15)
Pneumococcal vaccine^f^	25 (13)
Influenza vaccine^f^	66 (33)
Antibiotics prior to hospital admission^f^	90 (36)
Immunosuppressive drugs^g^	37 (14)

Note: Values are presented as No. (%) or median (25th–75th percentile). COPD, chronic obstructive pulmonary disease.

^a^Coronary heart disease, cerebrovascular disease and/or peripheral artery disease.

^b^Eleven patients with rheumatoid arthritis, 1 patient with systemic lupus erythematosus, 6 patients with inflammatory bowel disease, 1 patient with autoimmune hepatitis, 3 patients with Sjogren’s disease, 14 patients with psoriasis (2 patients had 2 conditions).

^c^Central nervous disease and/or neuromuscular disease.

^d^One patient with antibody deficiency, 1 patient with HIV, 1 patient with heart transplant, 2 patients with kidney transplant, 1 patient with bone marrow transplant. Three patients had received chemotherapy and 2 had received radiation therapy within last 3 months.

^e^Travel abroad past 4 weeks.

^f^Vaccinated against pneumococcus within previous 10 years; vaccinated against influenza virus within previous 12 months; antibiotics within previous 3 months.

^g^Immunosuppressive drugs were defined as any use of systemic steroids, Azathioprine, TNF-alpha inhibitor, Cyclosporine, Cyclophosphamide and/or Methotrexate within previous 3 months.

### Microbiological etiology of CAP

A definite or probable etiology was established, by all methods, in 167 (63%) of 267 patients, 58 (22%) of whom diagnosis was definite. Bacterial etiology was established in 126 (47%) of patients; *S. pneumoniae* was most commonly detected (Table 2). Twenty-five patients (9%) had bacteremia; 21 (84%) of these cases had *S. pneumoniae* isolated of which 2 cases had additional findings of either *Haemophilus influenzae* or *Moraxella catarrhalis.* One case had both *H. influenzae* and *Haemophilus parainfluenzae* isolated from blood, and *Escherichia coli*, *Pseudomonas aeruginosa* and group A streptococci was isolated from one case each. There were 7 cases of legionellosis (3 females and 4 males; age range 41–72 years), all caused by *L. pneumophila* serotype 1. Six of these patients had a documented travel history within past 4 weeks from a Mediterranean country (5 cases) or Asia (1 case), and 1 patient was probably infected while working on a supply-boat in the North Sea. Viral etiology was established in 92 (34%) of 267 patients; influenza viruses were most frequently detected followed by rhinovirus (Table 3). One patient had *Pneumocystis jirovecii* detected in BAL by use of real-time PCR.

**Table 2:** **Bacterial findings and contribution of different methods to diagnostic yield in the study population**

**Pathogen**	**No. (%) of patients with positive findings** **(n = 267)**	**Blood culture** **(n = 267)**	**Pleural fluid culture** **(n = 14)**	**Urinary antigen test** **(n = 262)**	**BAL** **(n = 8)**	**Sputum sample for culture and/or** ***L. pneumophila*** **PCR** **(n = 165)** ^**a**^	**NP swab culture** **(n = 263)**	**PCR**		**Serology** **(n = 263)**
								**NP swab** **(n = 262)** ^**b**^	**OP swab** **(n = 262)** ^**c**^	
*Streptococcus pneumoniae*	81 (30)	21	-	24	-	4	10	6	16	NA
*Bordetella pertussis*	15 (6)	NA	NA	NA	NA	NA	NA	-	-	15
*Haemophilus influenzae*	14 (5)	1	-	NA	-	5	8	NA	NA	NA
*Mycoplasma pneumoniae*	10 (4)	NA	NA	NA	NA	NA	NA	7	1	2
*Chlamydophila pneumoniae*	7 (3)	NA	NA	NA	NA	NA	NA	-	2	5
*Legionella pneumophila*	7 (3)	NA	NA	7	NA	-	NA	NA	NA	NA
*Enterobacteriaceae* ^*d*^	6 (2)	2	-	NA	-	3	1	NA	NA	NA
*Moraxella catarrhalis*	5 (2)	-	-	NA	-	2	3	NA	NA	NA
*Miscellaneous* ^*e*^	3 (1)	1	1	NA	-	-	1	NA	NA	NA
*Haemophilus parainfluenzae*	2 (1)	1	-	NA	-	1	-	NA	NA	NA
Total^f^	126 (47)	26	1	31	-	15	23	13	19	22

Note: Data are number of patients, unless otherwise stated, whose infections were etiologically established by use of a particular method listed in descending order of specificity (NP vs. OP swab PCR depends on pathogen tested). Additional patients had etiology established by use of different methods; e.g., *S. pneumoniae* infection was established by use of urinary antigen test in 24 additional patients whose etiology was not established by use of blood culture etc. PCR: *S. pneumoniae* was detected by use of qPCR; and *L. pneumophila, M. pneumoniae, C. pneumoniae* and *B. pertussis* by use of real-time PCR. BAL, bronchoalveolar lavage; qPCR, real-time quantitative polymerase chain reaction; NP, nasopharynx; OP, oropharynx; NA, not applicable.

^a^Of 165 sputum samples, 73 were of good quality. One of these tested positive for *L. pneumophila* by use of real-time PCR (urinary antigen test in this case was also positive).

^b^Of 262 patient samples, 240 revealed valid results for qPCR detection of *S. pneumoniae,* and 259 for real-time PCR detection of *M. pneumoniae, C. pneumoniae* and *B. pertussis.*

^c^Of 262 patient samples, 238 revealed valid results for qPCR detection of *S. pneumoniae*, and 259 for real-time PCR detection of *M. pneumoniae, C. pneumoniae* and *B. pertussis.*

^d^Include either of the following: *E. coli, P. aeruginosa* or Enterobacter species.

^e^Blood culture, Group A streptococcus; Pleural fluid culture, *Prevotella* spp. and *Dialister pneumosintes* (isolated from the same patient); NP swab culture, Group A streptococcus.

^f^No. of patients does not add up to no. of pathogens because some patients had multiple pathogens detected: a total of 150 bacteria were detected in 126 patients.

**Table 3:** **Viral findings and contribution of different methods to diagnostic yield in the study population**

**Pathogen**	**No. (%) of patients with positive findings** **(n = 267)**	**Real-time PCR**		**Serology** **(n = 263)**
		**NP swab** **(n = 262)** ^**a**^	**OP swab** **(n = 262)** ^**b**^	
Influenza viruses^c^	40 (15)	15	3	22
Rhinovirus	32 (12)	27	5	NA
Parainfluenza viruses^d^	8 (3)	5	3	NA
Respiratory syncytial virus^e^	7 (3)	7	-	NA
Metapneumovirus	7 (3)	4	3	NA
Enterovirus	5 (2)	5	-	NA
Adenovirus	1 (0.4)	1	-	NA
Total^f^	92 (34)	64	14	22

Note: Data are number of patients, unless otherwise stated, whose infections were etiologically established by use of a particular method listed in descending order of specificity (NP vs. OP swab PCR are generally considered equally). Additional patients had etiology established by use of different methods; e.g., infection with influenza viruses was established by use of OP swab in 3 additional patients whose etiology was not established by NP swab etc. PCR, polymerase chain reaction; NP, nasopharynx; OP, oropharynx; NA, not applicable.

^a^Of 262 patient samples, 240 revealed valid results for detection of all viruses (except from influenza viruses of which 239 samples revealed valid results).

^b^Of 262 patient samples, 238 revealed valid results for detection of all viruses.

^c^Influenza A virus (13 cases of which 2 cases were H1N1 positive), Influenza B virus (5 cases). 1 case tested positive for both influenza A and B viruses by use of serology.

^d^Parainfluenza virus type 1 (1 case), type 2 (2 cases), type 3 (5 cases).

^e^Respiratory syncytial virus A (5 cases), 2 cases were undefined.

^f^No. of patients does not add up to no. of pathogens because some patients had multiple pathogens detected: a total of 100 viruses were detected in 92 patients.

### Microbial patterns in CAP patients with an established etiology

A total of 251 pathogens were detected in the samples from 167 patients, the distribution patterns of the microbial agents identified in these mono- and coinfected patients are presented in Table 4. Multiple pathogens were detected in the samples from 69 (41%) cases; 1 additional pathogen (copathogen) in 54 (32%), and 2 copathogens in 15 (9%) cases. *S. pneumoniae* was identified in 44 (64%) of the 69 coinfected cases. Also, ≥1 copathogen was detected in 44 (54%) of 81 episodes with *S. pneumoniae.* This copathogen was due to 1 additional bacterial agent in 15 (19%) of cases; and/or ≥1 viral agent in 36 (44%). Accordingly, 36 (82%) of the 44 coinfected *S. pneumoniae* episodes were attributed to ≥1 viral agent. A pure bacterial etiology was established in 75 (45%) of the 167 patients; and a pure viral etiology in 41 (25%). Viral–bacterial coinfections were established in 51 (31%) of patients; the most common combinations were *S. pneumoniae* with influenza viruses (16 of 51 patients, 31%) or rhinovirus (14 of 51, 27%) (Additional file 1: Table S1).

**Table 4:** **Distribution of single and multiple bacterial and viral agents detected in 167 adults with an etiologically established diagnosis of community-acquired pneumonia**

**Bacterial agents**					
	**Pure bacterial infections** **(n = 75)**		**Viral–bacterial coinfections** **(n = 51)**		**Total**
	**Only one**	**Plus other bacterium**	**Plus virus** ^**a**^	**Plus virus and other bacterium**	**N (%)**
*Streptococcus pneumoniae*	37	8	29	7	81 (49)
*Bordetella pertussis*	2	4	4	5	15 (9)
*Haemophilus influenzae*	2	5	5	2	14 (8)
*Mycoplasma pneumoniae*	7	3			10 (6)
*Chlamydophila pneumoniae*	5	1		1	7 (4)
*Legionella pneumophila*	2	3		2	7 (4)
*Enterobacteriaceae*	1	3	2		6 (4)
*Moraxella catarrhalis*	2		1	2	5 (3)
*Miscellaneous* ^*b*^	1	1		1	3 (2)
*Haemophilus parainfluenzae*	1	1			2 (1)
Subtotal cases with bacteria	60				126 (75)^c^
**Viral agents**					
	**Pure viral infections** **(n = 41)**		**Viral–bacterial coinfections** **(n = 51)**		**Total**
	**Only one**	**Plus other virus**	**Plus bacterium** ^**a**^	**Plus bacterium and other virus**	**N (%)**
Influenza viruses	15^d^	2	21	2	40 (24)
Rhinovirus	12	2	15	3	32 (19)
Parainfluenza viruses	3		4	1	8 (5)
Respiratory syncytial virus	3	2	2		7 (4)
Metapneumovirus	3		3	1	7 (4)
Enterovirus	2		1	2	5 (3)
Adenovirus				1	1 (0.6)
Subtotal cases with viruses	39				92 (55)^c^

Note: Drammen, Norway, January 2008–January 2011.

^a^One or two.

^b^Group A streptococcus; *Prevotella* spp.; *Dialister pneumosintes*.

^c^Patients with multiple agents detected were counted as one individual case.

^d^One patient was also coinfected with *Pneumocystis jirovecii*.

### Microbial yield of different diagnostic methods

Etiology was established by conventional methods (i.e., bacterial cultures, urinary antigen assays and serology) in 127 (48%) of 267 cases; ≥1 bacterial agent was found in 107 (40%); and influenza viruses in 37 (14%). At least 1 copathogen was detected in 29 (11%) of cases. PCR alone provided etiology in 102 (38%) of cases; ≥1 bacterial agent was found in 51 (19%); ≥1 viral agent in 72 (27%); and ≥1 copathogen in 27 (10%). The total diagnostic yield improved by 15% (167 vs. 127 of cases) when PCR was added to conventional methods. Eleven different types of viruses were detected by PCR.

**Figure 2:**
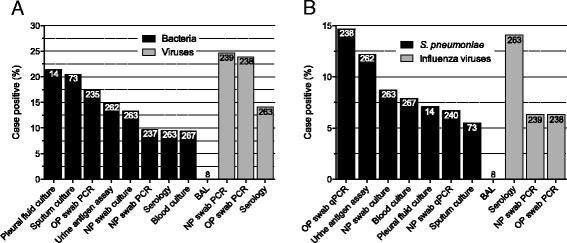
**Diagnostic yields of different microbiological methods.** Bars are percentages of cases with a positive test relative to the number of cases with a valid test, *n* (numbers inside the bars). Sputum culture: good-quality sputum by microscopy. **A**: Detection of bacteria and viruses, regardless of detection spectrum. **B**: Detection of *S. pneumoniae* and influenza viruses. OP, oropharynx; NP, nasopharynx; *S. pneumoniae, Streptococcus pneumoniae*; BAL, bronchoalveolar lavage; PCR, polymerase chain reaction.

The diagnostic yields obtained by the diverse methods are shown (Figure 2A and B). The corresponding contribution of each method for establishing bacterial or viral etiology in the study population is illustrated in Table 3 and Table 4, respectively. A pairwise comparison of the diagnostic yield obtained by each technique revealed interesting findings (Additional file 1: Table S2). First, OP swabs revealed significantly more positive results (34/235 positive, 14.5%) than NP swabs (14/235, 6.0%) for detection of *S. pneumoniae* by qPCR*, P* < .001*.* Secondly, for detection of *B. pertussis* serology exhibited significantly more positive results (13/256, 5.1%) than real-time PCR, in both NP and OP swabs (both 0/256, 0.0%) *P* = .001. Similar results were recorded for detection of influenza viruses by serology (34/236, 14.4%) compared to real-time PCR in NP swabs (15/236, 6.4%) *P* < .001; and serology (34/235, 14.5%) compared to real-time PCR in OP swabs (15/235, 6.4%) *P* < .001. Thirdly, there was no significant difference between sputum culture and NP swab culture for detection of bacteria (Figure 2A). Of note, sputum samples were collected from 165 (62%) patients; of these were only 73 (44%) deemed valid for culture by microscopy.

### Microbial yield in patients with complete sample collection

**Figure 3:**
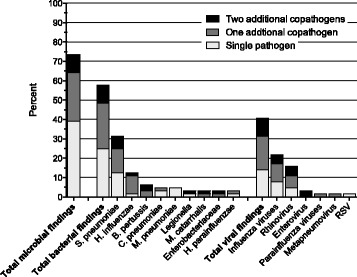
**Microbial findings in 64 cases with complete sampling and the proportion of coinfections.** *S. pneumoniae, Streptococcus pneumoniae; H. influenzae, Haemophilus influenzae; B. pertussis, Bordetella pertussis; C. pneumoniae, Chlamydophila pneumoniae; M. pneumoniae, Mycoplasma pneumoniae; M. catarrhalis, Moraxella catarrhalis; H. parainfluenzae, Haemophilus parainfluenzae.*

Microbial etiology was established in 47 (73%) of 64 patients with complete sample collection (Figure 3). The total diagnostic yield improved by 8% (47 vs. 42 of 64 cases, *P* = .44) when PCR was added to conventional methods; 5% (37 vs. 34) in cases with bacterial etiology; and 19% (26 vs. 14, *P* = .04) in cases with viral etiology. Cases with detection of ≥1 copathogen improved by 16% (22 vs. 12). Of 20 (31%) patients in whom *S. pneumoniae* was identified, 12 (60%) had ≥1 copathogen detected. This copathogen was due to ≥1 viral agent in 9 (45%) of patients. Etiology was established in 34 (79%) of 43 patients with complete sampling and who had not been prescribed antibiotics prior to hospital admission (not shown).

### Seasonality

**Figure 4:**
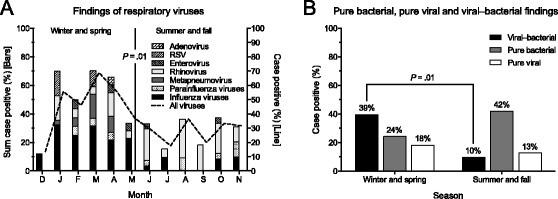
**Seasonal variations of microbial findings in patients with CAP during a 3-year period. A**: Monthly distribution of viral findings. Each segment in the stacked bars represents the proportion of cases with a positive test relative to those with a valid test for detection of a specific virus. At least one test (PCR or serology) was demanded for a valid detection of influenza viruses (n = 266), whereas only PCR for all other viruses (n = 243). Continuous line shows the proportion of cases with a positive test of any virus relative to those with a valid test for the complete detection of viruses (n = 231). **B**: Pure bacterial, pure viral and viral–bacterial findings in a subset of 64 patients with complete samples collected. RSV, Respiratory syncytial virus; CAP, community-acquired pneumonia.

Adult CAP patients with viral findings showed marked seasonal variations during the study period; the proportion of cases with positive findings was significantly higher in the winter and spring compared to summer and fall (45% vs. 28%, *P* = .01) (Figure 4A). Annually outbreaks of influenza viruses and *S. pneumoniae* contributed to the increase of these findings during the cold half of the year (Additional file 1: Figure S1). Only 2 cases of influenza A (H1N1) virus infection were detected, although the H1N1 pandemic reached our region in the middle of the inclusion period, May 2009. Detection rates of viral–bacterial coinfections in patients with CAP also varied considerably from month to month (range 0%–60%), with peaks in January and February (not shown); the proportion of viral–bacterial coinfections was significantly higher in the winter and spring compared to summer and fall (39% vs. 10%, *P* = .01) (Figure 4B). During winter and spring, viral–bacterial coinfections were more frequently detected than either pure infection alone.

## Discussion

This study is the first to describe etiology of CAP in hospitalized adults in Norway using conventional methods and PCR. The main results were: Firstly, etiology was established in the majority of patients, especially when samples were complete and no antibiotics were prescribed prior to hospital admission. Furthermore, PCR improved diagnostic yield, particularly in diagnosing viral infections. Secondly, *S. pneumoniae* and respiratory viruses (mainly influenza viruses and rhinovirus) were most commonly detected, usually with copathogens. Viruses were detected more frequently during winter and spring, which was also the time period they occurred most commonly in combination with bacteria. Thirdly, OP swabs gave more positive results than NP swabs for qPCR detection of *S. pneumoniae*; and serology gave more positive results than real-time PCR for detection of *B. pertussis* and influenza viruses.

Establishing a microbiological diagnosis for patients with CAP is challenging. As is the case in any study of samples not obtained through invasive techniques and with quantitation, the link between presence and causal is still quite unclear. However, in this study we applied a vast array of available tests to determine the presence of known pathogens, including those only identified by real-time PCR, following recommendations for interpretation applicable to our region. The diagnostic yield of 63% is consistent with results of other studies, although reports vary considerably from 39% to 76% [11-16,27-31]. These variations may be attributable to differences in the epidemiology of pathogens, study population, diagnostic methods and available patient specimens. The high diagnostic yield that was achieved in the complete sampling group underscores that etiology, nowadays, can be established in the majority of CAP patients if multiple techniques are applied and sample collection is optimized.

The prevalence of *S. pneumoniae*, other “typical” bacteria and *Legionella pneumophila*, which almost exclusively occurred in “imported” cases, are in line with previous studies from Northern-Europe [7,11] and USA [32]. *C. pneumoniae* was expectedly uncommon [7,11]. The relatively high frequency of *B. pertussis* is probably explained by an ongoing epidemic [33], and the low frequency of *M. pneumoniae* due to timing; the study was conducted between two 5–7 years epidemic cycles [34].

The potential role of viruses in causing pneumonia, both as the sole pathogen as well as in association with bacteria, is debated. Furthermore, the validity of upper-airway samples to establish an etiologic diagnosis is controversial. However, the impacts of viruses per se on adult CAP are increasingly being recognized [35-38]. In line with other studies [11,28,29], we identified viruses by all methods in 34% of all cases and in 41% of cases with complete sampling. In contrast, conventional methods identified only 14% of all cases, which confirms the increased sensitivity of PCR reported by others [11,16,39]. As expected, the occurrence of adult CAP with viral findings varied considerably during the year, peaking in March at ~70%, and was significantly higher during winter and spring compared to summer and fall. Influenza viruses were most frequently detected; 14% by serology compared to 6% by PCR, the difference between the two methods being statistically significant—and no more than 2 cases of influenza A (H1N1) virus infection were detected by PCR—illustrating the substantial value of serology in epidemiological studies. However, serology is generally not recommended for clinical decision making due to low specificity, which in our study may have led to an overestimation of numbers since a probable diagnosis was not only based on seroconversion but for some patients on high titer in single sample. Interestingly there was no increase in CAP admissions due to influenza viruses during the 2009 H1N1 pandemic, indicating that influenza viruses are major pathogens regularly occurring in patients hospitalized with CAP irrespective of the H1N1 pandemic. The low diagnostic yield of influenza viruses by PCR technique also underscores the clinical problem with false negative PCR influenza tests, and may suggest that sampling from both the upper and lower respiratory tract should be performed, at least in severely affected individuals [40]. Another explanation may be that the PCR influenza test is more likely to be negative later in the course of a secondary pneumonia to influenza virus infection leading to hospitalization.

Coinfections have previously been reported to account for 4% to 30% of adult CAP [28,30]. In our study, more than one pathogen was found in 26% of all patients and in 34% of patients with complete sampling. In agreement with recent studies, *S. pneumoniae* was the most common bacterial agent associated with coinfections, and a copathogen was found in more than one-half of the cases with the majority of pathogens being viral agents [11,12,14,41], usually influenza viruses or rhinovirus. One of our important observations, applying comprehensive and complete diagnostics, was an exceptionally high detection rate of viral–bacterial coinfections in the course of the winter season, peaking at 60% in January and February. This number is considerably higher than the 39% recently reported [40], indicating that seasonal activity patterns of respiratory pathogens also impacts on the occurrence of adult CAP with viral–bacterial coinfections, which appear to be the dominant cause of pneumonia during those times.

Good-quality sputum culture is often difficult to obtain and reported yields are varying [1]. In our study, sputum samples were obtained from 62% of patients; of those obtained were 56% judged as inadequate for culture, and of those cultured revealed 21% a positive result. NP swab cultures were obtained from 99% of patients; only 13% of these revealed a positive result. Our concordance analyses indicate, however, that the two methods are complementary.

PCR improved the yield by 8–15% when combined with conventional methods, proving that PCR is a valuable tool in addition to conventional methods. However, since serological tests have little clinical impact in the acute situation, our findings underscore the need for further improvements. Our results, using flocked swabs, indicate quite equal effectiveness of NP and OP sampling for real-time PCR detection of viruses and the concordance rate between the two specimens was good (~90%). These results are in contrast to those from two previous reports [29,42] that observed superiority of NP sampling, one of the studies using flocked swabs [42]. To our knowledge, no study comparing NP and OP sampling for qPCR detection of *S. pneumoniae* in adult CAP has yet been published: In line with the findings of Principi et al. [26], we found that OP swabs were significantly more effective than NP swabs for qPCR detection of *S. pneumoniae.* However, it is not known whether these might actually represent false-positives as, according to our analyses, OP swabs showed poorer specificity (90.2%) compared to NP swabs (96.8%) (Additional file 1). Furthermore, NP swabs were not assessed by direct fluorescent antigen for specimen adequacy prior to testing. However, excellent specimen adequacy of NP flocked swabs has recently been reported [43]. Nevertheless, due to the lack of diagnostic gold standards, accuracy of such tests is difficult to assess and more research is needed to confirm this finding.

Certain limitations concern data collection and analyses. Firstly, the requirement of rectal temperature >38.0°C may exclude predominantly elderly patients with CAP, who remain afebrile, and hence contribute to explain the low number of nursing home residents in the study. Secondly, some patients who met the inclusion criteria were not enrolled in the study and the complete microbial sampling could not be applied in every patient. We have, however, no reason to suspect these factors to induce systematic selection bias. Thirdly, not all sputum and NP culture samples were taken before administration of antibiotics, which influences the validity of the microbial etiology patterns found and the comparison of diagnostic tests. Fourthly, specificity issues have been raised regarding the *ply* gene used, which has been identified in *Streptococcus mitis*. However, we used quantitative technique, and any DNA amount of *S. mitis* would probably have been low, at least in NP samples. Furthermore, we did not include a control group to determine the prevalence of adult pneumococcal nasopharyngeal carriage. The frequency of carriage in Norway hardly differs appreciably from the 4% reported from Sweden [44]. Also, we used a *C*_q_ cut-off value of 10^5^ cfu/mL to distinguish colonization from true infection [25]. Because we did not include control subjects we were unable to investigate the significance of the proportion of certain viruses, in particular rhinovirus, among patients with CAP. Finally, we included 48 (18%) patients with immunosuppression (i.e., primary or acquired immunodeficiency, active malignancy, immunosuppressive drugs) that may have an impact on the etiological results. However, this proportion of immunocompromized patients in our study does not exceed 25% that probably would have hampered comparison of etiological results with other studies from which such patients have been excluded [7]. Moreover, inclusion of these patients may also be a strength of the study as they better reflect the population being referred to this local hospital. These limitations have been considered in the analyses and presentation of results.

## Conclusions

In conclusion, it was possible to establish etiology in 4 out of 5 CAP patients with the aid of PCR, which was particularly useful in diagnosing viral infections. The main pathogens identified were *S. pneumoniae* and viruses, most often influenza viruses and rhinovirus, usually detected with other agents. Viruses were more frequently detected during winter and spring, which was also the time period they occurred most commonly in combination with bacteria. Further research is needed to assess whether OP is superior to NP swabbing for qPCR detection of *S. pneumonia* in adults with CAP.

## Additional file

Additional file 1:
**Methods.** Details of serological methods. Details of real-time PCR methods and pneumococcal qPCR assay. Determination of the detection range of the pneumococcal qPCR assay and the cut-off quantification cycle (*C*_q_) value corresponding to 10^5^ cfu/mL. Accuracy of the pneumococcal qPCR assay. **Table S1**, **Table S2**, **Figure S1**, References.

## Abbreviations

CAP: Community-acquired pneumonia
PCR: Polymerase chain reaction
NP: Nasopharynx
OP: Oropharynx
BAL: Bronchoalveolar lavage
CFT: Complement fixation test
ELISA: Enzyme-linked immunosorbent assay
PT-IgG: Serum IgG against pertussis toxin IgG
qPCR: real-time quantitative PCR
Ply: Pneumolysin
RT-PCR: Reverse transcription PCR
*C*_q_: quantification cycle
CFU: Colony-forming units
SD: Standard deviation
COPD: Chronic obstructive pulmonary disease
NT: Not tested
ID: Identification
CRF: Case record form
RSV: Respiratory syncytial virus
