# Supplementary material for: Etiology of community-acquired pneumonia and diagnostic yields of microbiological methods: a 3-year prospective study in Norway
Source: BMC Infect Dis. 2015 Feb 15;15:64. doi: 10.1186/s12879-015-0803-5 (PMC4334764; doi:10.1186/s12879-015-0803-5)
Supplement: Additional file 1: — Methods. Details of serological methods. Details of real-time PCR methods and pneumococcal qPCR assay. Determination of the detection range of the pneumococcal qPCR assay and the cut-off quantification cycle (C q) value corresponding to 105 cfu/mL. Accuracy of the pneumococcal qPCR assay. Table S1, Table S2, Figure S1, References. [file 12879_2015_803_MOESM1_ESM.docx]

**SUPPLEMENTARY DATA**

**Manuscript title:**

Etiology of community-acquired pneumonia and diagnostic yields of microbiological methods: A 3-year prospective study in Norway

**Authors:**

Jan C. Holter, MD; Fredrik Müller, MD, PhD; Ola Bjørang, MSc; Helvi H. Samdal, MD;

Jon B. Marthinsen, MD; Pål A. Jenum, MD, PhD; Thor Ueland, PhD; Stig S. Frøland, MD, PhD;

Pål Aukrust, MD, PhD; Einar Husebye, MD, PhD; Lars Heggelund, MD, PhD.

**Methods**

**Details of serological methods**

Complement fixation test (CFT) was performed for determination of complement-fixing antibodies against influenza A and B viruses (SERION CFT reagents, Institut Virion\Serion GmbH); and a qualitative and quantitative enzyme-linked immunosorbent assay (SERION ELISA *classic* Bordetella pertussis Toxin IgA and IgG test) was used for the detection of *B. pertussis* toxin (PT-IgG). If paired samples were available, serologic testing for *M. pneumoniae* and *C. pneumoniae* were performed by CFT (SERION CFT reagents, Institut Virion\Serion GmbH), otherwise by immunoassays for IgM detection (NOVITEC Mycoplasma and NOVITEC Chlamydia pneumoniae; HiSS Diagnostics, Freiburg, Germany) on acute- or convalescent-phase sample. The ELISA tests were performed on Best 2000/1DXC-1267. All paired samples were run in parallel using same lots and plates.

**Details of real-time PCR methods and pneumococcal qPCR assay**

Single-agent assays were performed consecutively by real-time PCR for detection of *M. pneumoniae* (ABI 7500 Fast, Applied Biosystems^®^, Rotkreuz, Switzerland); primers TTCTTCAGGCTCAGGTCAATCTG and CCGTCACTCGTGCTTGGTA, TaqMan-probe FAM- ACTGCCCCACAAGCC (targeting the P1 adhesin gene); and for detection of *C. pneumoniae* [1]*, B. pertussis* [2]*, L. pneumophila* [3] and *P. jirovecii* [4] using LightCycler 2.0^®^ (Roche Diagnostics, Basel, Switzerland).

Detection of *S. pneumoniae* and respiratory viruses were performed retrospectively. For detection and quantification of *S. pneumoniae*, we used primers specific for *ply* gene, probe sequences and TaqMan assay as described by Greiner *et al* [5]. Primers were obtained from Invitrogen (Life Technologies^TM^, US) and TaqMan probes from TIB MolBiol (TIB MolBiol, Berlin, Germany). With regard to respiratory viruses, four reactions were in-house multiplex real-time RT-PCR assays; influenza A [6] and B [7] viruses; parainfluenza viruses types 1 [8], 2 and 3 [9]; metapneumovirus [10] and rhinovirus [11]; and enterovirus [12] and respiratory syncytial virus (A and B) [13]. The other reactions were performed as in-house single assays; H1N1 real-time RT-PCR on influenza A virus positive samples [14]; and real-time PCR for the detection of adenovirus [15]. In each reaction Rnase P [6] was included as an internal control. In brief, automated genomic RNA and DNA extraction was performed with MagNa pure LC using the total nucleic acid isolation kit system according to the manufacturer’s protocol (Roche Diagnostics), cDNA was synthesized by using qScript^TM^ cDNA SuperMix (Quanta Biosciences). The real-time PCRs were carried out on the LightCycler^®^480II (Roche Diagnostics).

**Determination of the detection range of the pneumococcal qPCR assay and the cut-off quantification cycle (*C*_q_) value corresponding to 10^5^ cfu/mL**

To determine the detection range of the qPCR assay and to assess the corresponding *C*_q_ value, a standard curve for *S. pneumoniae* was generated as follows: The *S. pneumoniae* reference strain ATCC 49619 was inoculated onto horse blood agar and incubated at 37°C in aerobic atmosphere with 5% CO2 for 24 h. Bacteria were suspended in physiological saline at a density of 0.5 McFarland (Vitek Densichek, Biomerieux), representing approximately 10^8^ cfu/mL and 10-fold serial dilutions in physiological saline were prepared. The number of colony-forming units was determined by plating 10 μL and 100 μL of each dilution onto the agar plates with aerobic incubation overnight at 37°C. Two hundred μL of each dilution was used for DNA extraction and analyzed by qPCR in triplicates. The cut-off value of 10^5^ cfu/mL was derived from studies of quantitative sputum cultures related to blood culture results [16], in agreement with validation—and clinical—studies using the *ply* gene [17,18]. 10^5^ cfu/mL corresponded to a *C*_q_ value of 28.05, and this *C*_q_ value was applied as a cut-off value for the clinical samples.

**Accuracy of the pneumococcal qPCR assay**

In the absence of a sensitive reference standard, the accuracy of the *ply* qPCR assay to distinguish between pneumococcal and non-pneumococcal etiology in patients with CAP were calculated using a pooled diagnostic standard for pneumococcal pneumonia (i.e., demonstration of pneumococci by any conventional method; blood culture, pleural fluid culture, urinary antigen assay, sputum or NP culture). ROC analysis (not shown) showed good diagnostic accuracy for NP sampling (AUC, 0.80 95%CI [0.72–0.87]) and fair diagnostic accuracy for OP sampling (AUC, 0.74 [0.66–0.82]). The use of pneumococcal DNA *C*_q_ cut-off corresponding to ≥10^5^ cfu/mL resulted in higher sensitivity and lower specificity for OP sampling (sensitivity 31.5%, specificity 90.2%) than for NP sampling (18.5%, 96.8%).

**Supplementary Table 1 Single and multiple bacterial and viral agents detected in 167 adults with an etiologically established diagnosis of community-acquired pneumonia**

| Pure bacterial and pure viral infections | n (%) | Viral–bacterial coinfections | n (%) |
| --- | --- | --- | --- |
| **Pure bacterial (single)** | **60 (36)** | **Viral–bacterial (dual)** | **36 (22)** |
| *S. pneumoniae* | 37 (22) | *S. pneumoniae* plus: |  |
| *M. pneumoniae* | 7 (4) | Influenza viruses | 13 (8) |
| *C. pneumoniae* | 5 (3) | Rhinovirus | 8 (5) |
| *H. influenzae* | 2 (1) | Metapneumovirus | 2 (1) |
| *M. catarrhalis* | 2 (1) | Parainfluenza viruses | 1 (0.6) |
| *L. pneumophila* | 2 (1) | Respiratory syncytial virus | 1 (0.6) |
| *B. pertussis* | 2 (1) | *H. influenzae* plus: |  |
| Group A streptococcus | 1 (0.6) | Parainfluenza viruses | 2 (1) |
| *H. parainfluenzae* | 1 (0.6) | Influenza viruses | 1 (0.6) |
| *Enterobacteriaceae* | 1 (0.6) | Metapneumovirus | 1 (0.6) |
| **Pure viral (single)** | **38 (23)** | Rhinovirus | 1 (0.6) |
| Influenza viruses^a^ | 15 (9) | *B. pertussis* plus: |  |
| Rhinovirus | 12 (7) | Influenza viruses | 3 (2) |
| Metapneumovirus | 3 (2) | Respiratory syncytial virus | 1 (0.6) |
| Parainfluenza viruses | 3 (2) | *M. catarrhalis* plus: |  |
| Respiratory syncytial virus | 3 (2) | Influenza viruses | 1 (0.6) |
| Enterovirus | 2 (1) | *Enterobacteriaceae* plus: |  |
| Adenovirus | 0 (0) | Rhinovirus | 1 (0.6) |
| **Pure bacterial (dual)** | **15 (9)** | **Viral–bacterial (multiple)** | **15 (9)** |
| *S. pneumoniae* plus: |  | *S. pneumoniae* plus: |  |
| *H. influenzae* | 4 (2) | *M. catarrhalis* and rhinovirus | 2 (1) |
| *Enterobacteriaceae* | 1 (0.6) | *H. influenzae* and enterovirus | 1 (0.6) |
| *L. pneumophila* | 1 (0.6) | Group A streptococcus and influenza viruses | 1 (0.6) |
| *M. pneumoniae* | 1 (0.6) | *B. pertussis* and influenza viruses | 1 (0.6) |
| *B. pertussis* | 1 (0.6) | *B. pertussis* and rhinovirus | 1 (0.6) |
| *L. pneumophila* plus: |  | *C. pneumoniae* and parainfluenza viruses | 1 (0.6) |
| *B. pertussis* | 2 (1) | Influenza viruses and rhinovirus | 1 (0.6) |
| *Enterobacteriaceae* plus: |  | Parainfluenza viruses and adenovirus | 1 (0.6) |
| *M. pneumoniae* | 1 (0.6) | Metapneumovirus and rhinovirus | 1 (0.6) |
| *C. pneumoniae* | 1 (0.6) | Rhinovirus and enterovirus | 1 (0.6) |
| *H. influenzae* plus: |  | *L. pneumophila* plus: |  |
| *H. parainfluenzae* | 1 (0.6) | *B. pertussis* and influenza viruses | 1 (0.6) |
| *M. pneumoniae* plus: |  | *B. pertussis* and rhinovirus | 1 (0.6) |
| *B. pertussis* | 1 (0.6) | *H. influenzae* plus: |  |
| *D. pneumosintes* plus: |  | *B. pertussis* and rhinovirus | 1 (0.6) |
| *Prevotella* spp. | 1 (0.6) | *Enterobacteriaceae* plus: |  |
| **Pure viral (dual)** | **3 (2)** | Influenza viruses and enterovirus | 1 (0.6) |
| Influenza viruses *plus:* |  | … | … |
| Rhinovirus | 1 (0.6) | … | … |
| Respiratory syncytial virus | 1 (0.6) | … | … |
| Rhinovirus *plus:* |  | … | … |
| Respiratory syncytial virus | 1 (0.6) | … | … |

Note: *S. pneumoniae, Streptococcus pneumoniae; M. pneumoniae, Mycoplasma pneumoniae; C. pneumoniae, Chlamydophila pneumoniae; H. influenzae, Haemophilus influenzae; M. catarrhalis, Moraxella catarrhalis; L. pneumophila, Legionella pneumophila; B. pertussis, Bordetella pertussis; H. parainfluenzae, Haemophilus parainfluenzae; D. pneumosintes, Dialister pneumosintes.*^a^ One patient was also coinfected with *Pneumocystis jirovecii*.

**Supplementary Table 2 Pairwise comparison of diagnostic yields between different techniques used for the detection of respiratory pathogens in patients with community-acquired pneumonia**

| Pathogen | Detection rate, % (No. positive/No. of cases with valid tests) | | | | | | | *P* | Agreement, % | Kappa |
| --- | --- | --- | --- | --- | --- | --- | --- | --- | --- | --- |
|  | Blood culture | Urinary antigen test | Sputum culture | NP swab culture | NP swab PCR | OP swab PCR | Serology |  |  |  |
| Bacteria^a^ |  |  | 20.5 (15/73) | 15.1 (11/73) |  |  |  | .52 | 69.9 | NC |
| *S. pneumoniae* | 8.0 (21/262) | 12.2 (32/262) |  |  |  |  |  | .10 | 85.9 | 0.23 |
| *S. pneumoniae* |  |  | 5.5 (4/73) | 9.6 (7/73) |  |  |  | .51 | 87.7 | 0.12 |
| *S. pneumoniae* |  |  | 5.9 (4/68) |  | 5.9 (4/68) |  |  | 1.00 | 91.1 | 0.20 |
| *S. pneumoniae* |  |  | 6.1 (4/66) |  |  | 15.2 (10/66) |  | .07 | 87.9 | 0.37 |
| *S. pneumoniae* |  |  |  | 9.2 (22/239) | 6.7 (16/239) |  |  | .31 | 90.0 | 0.32 |
| *S. pneumoniae* |  |  |  | 9.3 (22/237) |  | 14.8 (35/237) |  | .07 | 81.9 | 0.15 |
| *S. pneumoniae* |  |  |  |  | 6.0 (14/235) | 14.5 (34/235) |  | < .001 | 87.2 | 0.32 |
| *M. pneumoniae* |  |  |  |  | 2.7 (7/259) | 2.3 (6/259) |  | 1.00 | 98.8 | 0.76 |
| *M. pneumoniae* |  |  |  |  | 2.3 (6/256) |  | 1.2 (3/256) | .38 | 98.0 | 0.44 |
| *M. pneumoniae* |  |  |  |  |  | 2.3 (6/256) | 1.2 (3/256) | .38 | 98.0 | 0.44 |
| *C. pneumoniae* |  |  |  |  | 0.0 (0/259) | 0.8 (2/259) |  | .56 | 99.2 | NC |
| *C. pneumoniae* |  |  |  |  | 0.0 (0/256) |  | 2.3 (6/256) | .06 | 97.7 | NC |
| *C. pneumoniae* |  |  |  |  |  | 0.8 (2/256) | 2.3 (6/256) | .22 | 97.7 | 0.24 |
| *B. pertussis* |  |  |  |  | 0.0 (0/259) | 0.0 (0/259) |  | NC | 100.0 | NC |
| *B. pertussis* |  |  |  |  | 0.0 (0/256) |  | 5.1 (13/256) | .001 | 94.9 | NC |
| *B. pertussis* |  |  |  |  |  | 0.0 (0/256) | 5.1 (13/256) | .001 | 94.9 | NC |
| Respiratory viruses^b^ |  |  |  |  | 23.5 (55/234) | 24.4 (57/234) |  | .84 | 89.7 | 0.72 |
| Influenza viruses |  |  |  |  | 6.0 (14/234) | 6.4 (15/234) |  | 1.00 | 97.9 | 0.82 |
| Influenza viruses^c^ |  |  |  |  | 6.4 (15/236) |  | 14.4 (34/236) | < .001 | 89.4 | 0.44 |
| Influenza viruses^d^ |  |  |  |  |  | 6.4 (15/235) | 14.5 (34/235) | < .001 | 90.2 | 0.49 |

Note: Missing data were excluded from the analysis. *S. pneumoniae* was detected by use of qPCR; and *M. pneumoniae, C. pneumoniae, B. pertussis* and respiratory viruses by use of real-time PCR. NC, not calculated; *S. pneumoniae, Streptococcus pneumoniae*; *M. pneumoniae, Mycoplasma pneumoniae; C. pneumoniae, Chlamydophila pneumoniae; B. pertussis, Bordetella pertussis;* NP, nasopharynx; OP, oropharynx; PCR, polymerase chain reaction; qPCR, real-time quantitative PCR.

^a^ Any bacteria growth.

^b^ Any of the following: influenza viruses, rhinovirus, parainfluenza viruses, respiratory syncytial virus, metapneumovirus, enterovirus or adenovirus.

^c^ If only seropositive–high titer tests were included, the numbers were: 6.4 (15/236) vs. 5.1 (12/236), *P* = .69, Agreement 89%, Kappa 0.02.

^d^ If only seropositive–high titer tests were included, the numbers were: 6.4 (15/235) vs. 5.1 (12/235), *P* = .66, Agreement 91%, Kappa 0.18.

**Supplementary figure 1 Seasonal distribution of CAP patients with influenza viruses and *S. pneumoniae* during a 3-year study period.** A total of 266 patients were tested for influenza A and B viruses by at least one method (PCR and/or serology). In Norway, the first 2 cases of 2009 pandemic influenza A (H1N1) virus infection were reported on May 9. The number of reported cases reached a peak in November and declined until December before WHO, on August 10, 2010, announced that the H1N1 pandemic had moved into the post-pandemic period. Only 2 cases of influenza A (H1N1) virus infection were detected in our hospital during the study period (not shown).

The 7-valent pneumococcal conjugate vaccine was introduced in the childhood immunization program in 2006. Due to herd effect, a decrease in the overall invasive pneumococcal incidence was observed among the elderly in Norway (> 50 years of age) until 2009, and was relatively stable through the remainder of the study period [19]. Thus, a reduction in our data during this period was not expected (a total of 267 patients were tested by at least one detection method for *S. pneumoniae*). Detection rates of influenza viruses and *S. pneumoniae* among hospitalized adult CAP patients appeared to follow normal seasonal variation with peaks in the cold seasons. *S. pneumoniae, Streptococcus pneumoniae*.

**References**

1. Mygind T, Birkelund S, Falk E, Christiansen G: **Evaluation of real-time quantitative PCR for identification and quantification of Chlamydia pneumoniae by comparison with immunohistochemistry**. *J Microbiol Methods* 2001, **46**(3):241-251.

2. Kosters K, Reischl U, Schmetz J, Riffelmann M, Wirsing von Konig CH: **Real-time LightCycler PCR for detection and discrimination of Bordetella pertussis and Bordetella parapertussis**. *J Clin Microbiol* 2002, **40**(5):1719-1722.

3. Reischl U, Linde HJ, Lehn N, Landt O, Barratt K, Wellinghausen N: **Direct detection and differentiation of Legionella spp. and Legionella pneumophila in clinical specimens by dual-color real-time PCR and melting curve analysis**. *J Clin Microbiol* 2002, **40**(10):3814-3817.

4. Larsen HH, Masur H, Kovacs JA, Gill VJ, Silcott VA, Kogulan P, Maenza J, Smith M, Lucey DR, Fischer SH: **Development and evaluation of a quantitative, touch-down, real-time PCR assay for diagnosing Pneumocystis carinii pneumonia**. *J Clin Microbiol* 2002, **40**(2):490-494.

5. Greiner O, Day PJ, Bosshard PP, Imeri F, Altwegg M, Nadal D: **Quantitative detection of Streptococcus pneumoniae in nasopharyngeal secretions by real-time PCR**. *J Clin Microbiol* 2001, **39**(9):3129-3134.

6. **WHO collaborating Centre for Influenza at CDC (2009) CDC protocol of real-time RTPCR for influenza A (H1N1)** [http://www.who.int/csr/resources/publications/swineflu/CDCRealtimeRTPCR_SwineH1Assay-2009_20090430.pdf]

7. Ward CL, Dempsey MH, Ring CJ, Kempson RE, Zhang L, Gor D, Snowden BW, Tisdale M: **Design and performance testing of quantitative real time PCR assays for influenza A and B viral load measurement**. *J Clin Virol* 2004, **29**(3):179-188.

8. Watzinger F, Suda M, Preuner S, Baumgartinger R, Ebner K, Baskova L, Niesters HG, Lawitschka A, Lion T: **Real-time quantitative PCR assays for detection and monitoring of pathogenic human viruses in immunosuppressed pediatric patients**. *J Clin Microbiol* 2004, **42**(11):5189-5198.

9. Echevarria JE, Erdman DD, Swierkosz EM, Holloway BP, Anderson LJ: **Simultaneous detection and identification of human parainfluenza viruses 1, 2, and 3 from clinical samples by multiplex PCR**. *J Clin Microbiol* 1998, **36**(5):1388-1391.

10. Maertzdorf J, Wang CK, Brown JB, Quinto JD, Chu M, de Graaf M, van den Hoogen BG, Spaete R, Osterhaus AD, Fouchier RA: **Real-time reverse transcriptase PCR assay for detection of human metapneumoviruses from all known genetic lineages**. *J Clin Microbiol* 2004, **42**(3):981-986.

11. Deffernez C, Wunderli W, Thomas Y, Yerly S, Perrin L, Kaiser L: **Amplicon sequencing and improved detection of human rhinovirus in respiratory samples**. *J Clin Microbiol* 2004, **42**(7):3212-3218.

12. Glimaker M, Johansson B, Olcen P, Ehrnst A, Forsgren M: **Detection of enteroviral RNA by polymerase chain reaction in cerebrospinal fluid from patients with aseptic meningitis**. *Scand J Infect Dis* 1993, **25**(5):547-557.

13. Brittain-Long R, Nord S, Olofsson S, Westin J, Anderson LM, Lindh M: **Multiplex real-time PCR for detection of respiratory tract infections**. *J Clin Virol* 2008, **41**(1):53-56.

14. **Robert Koch Institut. TaqMan real-time PCR zur Detektion von porcinen Influenza A/H1N1-Viren** [http:/http://www.rki.de/cln 179/nn 200120/DE/Content/InfAZ/I/Influenza/IPV/Schweinegrippe PCR.html]

15. Heim A, Ebnet C, Harste G, Pring-Akerblom P: **Rapid and quantitative detection of human adenovirus DNA by real-time PCR**. *J Med Virol* 2003, **70**(2):228-239.

16. Kalin M, Lindberg AA: **Diagnosis of pneumococcal pneumonia: a comparison between microscopic examination of expectorate, antigen detection and cultural procedures**. *Scand J Infect Dis* 1983, **15**(3):247-255.

17. Kais M, Spindler C, Kalin M, Ortqvist A, Giske CG: **Quantitative detection of Streptococcus pneumoniae, Haemophilus influenzae, and Moraxella catarrhalis in lower respiratory tract samples by real-time PCR**. *Diagn Microbiol Infect Dis* 2006, **55**(3):169-178.

18. Johansson N, Kalin M, Tiveljung-Lindell A, Giske CG, Hedlund J: **Etiology of community-acquired pneumonia: increased microbiological yield with new diagnostic methods**. *Clin Infect Dis* 2010, **50**(2):202-209.

19. Steens A, Bergsaker MA, Aaberge IS, Ronning K, Vestrheim DF: **Prompt effect of replacing the 7-valent pneumococcal conjugate vaccine with the 13-valent vaccine on the epidemiology of invasive pneumococcal disease in Norway**. *Vaccine* 2013, **31**(52):6232-6238.
